# Integrated Transcriptomics and Metabolomics Analysis Promotes the Understanding of Adventitious Root Formation in *Eucommia ulmoides* Oliver

**DOI:** 10.3390/plants13010136

**Published:** 2024-01-03

**Authors:** Qingxin Du, Kangkang Song, Lu Wang, Lanying Du, Hongyan Du, Bin Li, Haozhen Li, Long Yang, Yan Wang, Panfeng Liu

**Affiliations:** 1 State Key Laboratory of Tree Genetics and Breeding, Research Institute of Non-Timber Forestry, Chinese Academy of Forestry, Zhengzhou 450003, China; duqingxin20102325@163.com (Q.D.); wanglu181716@163.com (L.W.); dly371@126.com (L.D.); dhy515@caf.ac.cn (H.D.); 2 College of Forestry, Nanjing Forestry University, Nanjing 210037, China; 3 College of Plant Protection and Agricultural Big-Data Research Center, Shandong Agricultural University, Tai’an 271018, China; kangkangsong234@163.com (K.S.); 2021110144@sdau.edu.cn (B.L.);; 4 State Forestry and Grassland Administration Key Laboratory of Silviculture in Downstream Areas of the Yellow River, Mountain Tai Forest Ecosystem Research Station of State Forestry and Grassland Administration, College of Forestry, Shandong Agricultural University, Tai’an 271018, China

**Keywords:** *Eucommia ulmoides* Oliver, adventitious root, hormone, phenylpropanoid biosynthesis, lncRNAs

## Abstract

As a primary approach to nutrient propagation for many woody plants, cutting roots is essential for the breeding and production of *Eucommia ulmoides* Oliver. In this study, hormone level, transcriptomics, and metabolomics analyses were performed on two *E. ulmoides* varieties with different adventitious root (AR) formation abilities. The higher JA level on the 0th day and the lower JA level on the 18th day promoted superior AR development. Several hub genes executed crucial roles in the crosstalk regulation of JA and other hormones, including F-box protein (*EU012075*), SAUR-like protein (*EU0125382*), LOB protein (*EU0124232*), AP2/ERF transcription factor (*EU0128499*), and CYP450 protein (*EU0127354*). Differentially expressed genes (DEGs) and metabolites of AR formation were enriched in phenylpropanoid biosynthesis, flavonoid biosynthesis, and isoflavonoid biosynthesis pathways. The up-regulated expression of *PAL*, *CCR*, *CAD*, *DFR*, and *HIDH* genes on the 18th day could contribute to AR formation. The 130 cis-acting lncRNAs had potential regulatory functions on hub genes in the module that significantly correlated with JA and DEGs in three metabolism pathways. These revealed key molecules, and vital pathways provided more comprehensive insight for the AR formation mechanism of *E. ulmoides* and other plants.

## 1. Introduction

*Eucommia ulmoides* Oliver is a perennial tree with important medicinal, industrial, and ecological values [1,2,3]. *E. ulmoides* is an herbal medicine and edible oil resource and is also a natural high-quality rubber resource with great developmental potentials [4,5,6]. Cutting propagation has the advantages of stable genetic characteristics, a short seedling-raising period, simple operation, and low cost [7]. As a dioecious plant, the propagation of *E. ulmoides* mainly depends on cutting to speed up the growth process and maintain excellent characters in the genetic breeding. The formation of adventitious roots (AR) is closely related to the survival of cuttings [8,9]. The improvement of AR formation ability is essential and will greatly promote the breeding and production of *E. ulmoides*.

Plant hormones play an indispensable role in AR formation [10,11]. Jasmonic (JA) acts with auxin and other phytohormones through sophisticated crosstalk during AR development [12,13]. JA might enhance auxin synthesis in the induction stage to promote AR formation [14]. The reduction of AR numbers in JA-deficient cuttings indicated that JA played an active role in the AR formation of wild-type petunia [15]. JA could improve the biomass, total phenolic content, and antioxidant activity of AR of *Acmella radicans* cultured in ahake flasks [16]. Notably, some studies have denoted that low concentrations of methyl jasmonate (MeJA) and high concentrations of MeJA promoted and inhibited AR, respectively, which had opposite effects on AR formation [17,18]. In addition, other plant hormones can also regulate AR development through a series of crosstalk with JA and/or auxin, including ethylene, abscisic acid (ABA), salicylic acid (SA), and so on. JA could also mutually interfere with ethylene signaling to facilitate auxin-induced AR in dark-grown *Arabidopsis thaliana* seedlings [19]. In *Boehmeria nivea* L., JA could interact with ethylene to regulate the occurrence and number of AR [20]. ABA regulated indole-3-acetic acid (IAA) oxidase activity and the AR development of mung bean seedlings under cadmium stress [21]. Exogenous ABA inhibited the formation of AR at a dose of 0.25 μm or higher in *Arabidopsis* [22]. Low-concentration SA promoted AR development and altered the architecture of the root apical meristem [23]. The appropriate concentration of exogenous SA could improve IAA level and promote the AR formation of cucumber [24].

The genes related to AR formation have been found in many species, such as *AUXIN RESPONSE FACTOR* (*ARF*), *LATERAL ORGAN BOUNDARIES-DOMAIN (LBD*/*LOB*) [25], *APETALA2/Ethylene-responsive FACTOR* (*AP2*/*ERF*) [26], *SMALL AUXIN UP RNA* (*SAUR*), and *WUSCHEL-related HOMEOBOX* (*WOX*) [27]. *ARF6* and *ARF8* were promoters of AR development, whereas *ARF17* inhibited root formation [28]. *LBD16* and *LBD18* acting downstream of *ARF7* and *ARF19* participated in AR formation in *A. thaliana* [29]. The *PtaERF003* gene, belonging to the *AP2/ERF* family, enhanced the AR of poplar cuttings through the auxin signaling pathway [30]. SAUR15 could improve AR formation by activating H^+^-ATPase and auxin biosynthesis [31]. The heterologous overexpression of *LkWOX4* from *Larix kaempferi* in 84 K poplar altered the number and length of AR as well as remodeled the transcriptome, and the expressions of phytohormone-signaling genes (*ARF2*, *ARF3*, *ARF7*, and *ARF18*), rooting-associated transcription factors (*WOX5*, *LBD29*, and *SCR*), and root development-associated genes (*CYCD3*, *GRF1*, and *TAA1*) were modulated [32]. In addition, transcription factors, such as *MYB*, *NAC*, *MADS-box*, *WRKY*, *bHLH*, *bZIP*, and *GRAS* families, have also been found to mediate AR formation [33,34,35,36,37,38,39]. The phenylpropanoid metabolism plays a significant role in plant development and stress tolerance, and it is regulated by various plant hormones, including auxins, ethylene, JA, strigolactones (SL), and gibberellins (GA) [40,41]. In *Camellia sinensis*, genes related to AR development were significantly enriched in phenylpropanoid biosynthesis, plant hormone signal transduction, and flavonoid biosynthesis [42]. The high accumulation of metabolites related to phenylpropanoid and flavonoid biosynthesis pathways promoted the growth of AR of *Patycladus orientalis*, including caffeic acid and coniferyl alcohol, cinnamoyl-CoA, and isoliquiritigenin [43].

AR formation has been deeply studied in the model plants, including *A. thaliana* [44], *Oryza* [45], and *Populus* [46]. In *E. ulmoides*, the mechanism of the systematic regulation of AR formation by various hormones and key molecules remains elusive. The release of the high-quality genome of *E. ulmoides* provided an opportunity to deeply analyze the *E. ulmoides* AR formation at the molecular level [47,48]. In this study, physiological indexes, whole transcriptomics, and metabolomics during the AR development of *E. ulmoides* were comprehensively analyzed. The dynamic levels of IAA, ABA, JA, and SA hormones were examined. Weighted co-expression network analysis (WGCNA) was performed, combined with hormone traits and expressed genes. Differentially expressed genes (DEGs), differentially expressed metabolites (DEMs), and pathway enrichment were investigated by transcriptomic data and metabolomic data. The cis-acting long noncoding RNAs (lncRNAs) with potential regulatory functions were identified. This study provides abundant information for further research on the molecular mechanism during AR formation and has an important significance for the breeding and application of new varieties of *E. ulmoides*.

## 2. Results

### 2.1. Morphological Characteristics and Hormone Content during AR Development

**Figure 1:**
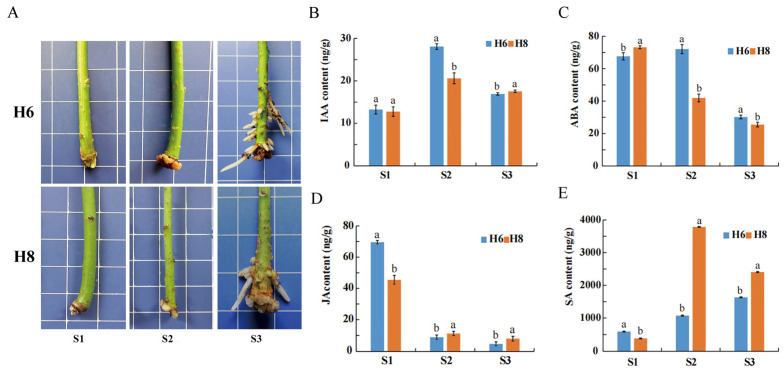
The morphological characteristics and hormone levels during the AR formation of two *E. ulmoides* varieties. (**A**) Morphological characteristics. (**B**) IAA level. (**C**) ABA level. (**D**) JA level. (**E**) SA level. S1, cutting on the 0th day. S2, cutting on the 18th day. S3, cutting on the 32nd day. Different letters denote significant differences at *p* < 0.05 level.

*E. ulmoides* Huazhong 6 (H6) had more AR numbers compared to *E. ulmoides* Huazhong 8 (H8) across three developmental stages, indicating obvious morphological differences (Figure 1A). This is consistent with our previous study on the average number of roots and average length of roots of H6 and H8 [8]. The hormone dynamic changes of H6 and H8 displayed similar rising or falling patterns in three stages. However, compared to H8, H6 exhibited significant differences in hormone levels in its roots at each stage (Figure 1B–E). In the S1 stage, H6 exhibited significantly higher levels of SA and JA compared to H8, and H6 had a significantly lower level of ABA than H8. In the S2 stage, H6 showed significantly higher levels of IAA and ABA compared to H8, and H6 had significantly lower levels of SA and JA than H8. In the S3 stage, H6 exhibited a significantly higher level of ABA compared to H8, and H6 had significantly lower levels of IAA, SA, and JA than H8. For each variety, the level of JA decreased, while the levels of IAA and SA increased from the S1 stage to the S2 stage.

### 2.2. The DEGs and KEGG Pathways Enrichment during AR Development

**Figure 2:**
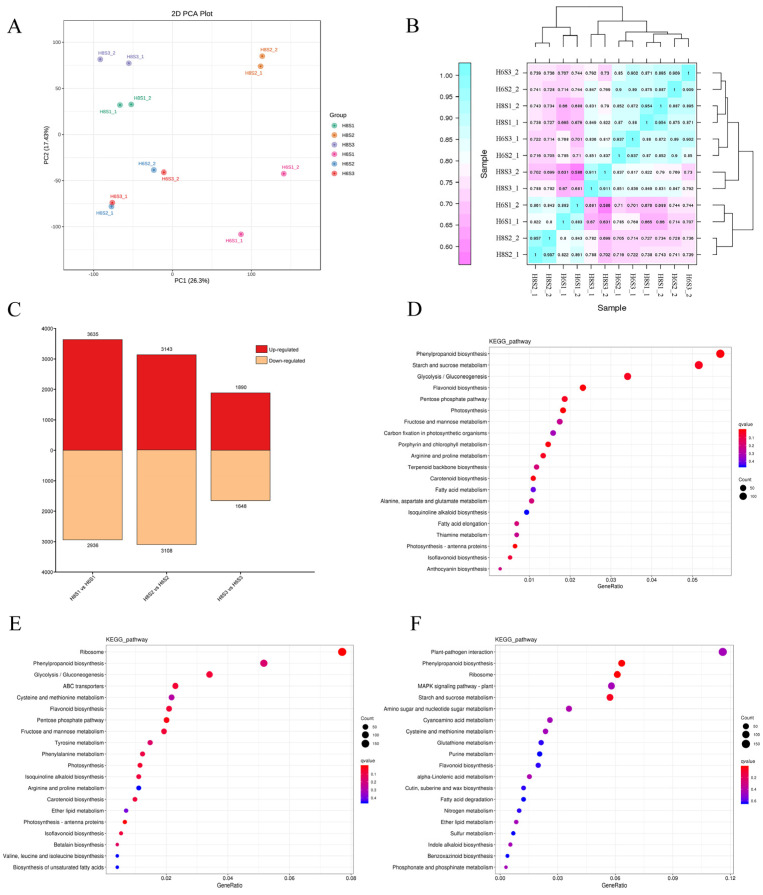
DEGs and KEGG enrichment analysis during AR development. (**A**) PCA of samples in three developmental stages. (**B**) Correlation analysis of samples. The scale bar represents the size of correlation. (**C**) DEGs of H8 and H6 in three developmental stages. (**D**) KEGG enrichment of DEGs in H8S1 vs. H6S1. (**E**) KEGG enrichment of DEGs in H8S2 vs. H6S2. (**F**) KEGG enrichment of DEGs in H8S3 vs. H6S3. The q-value is *p*-value after multiple hypothesis testing corrections based on false discovery rate measures.

The whole transcriptome strand-specific RNA sequencing of cuttings generated a total of 205.60 Gb clean bases (Q30 > 94.09%), with at least 15.67 Gb from each sample (Table S1). After mapping the clean reads to the *E. ulmoides* genome, 34,974 transcripts were finally obtained. In the principal component analysis (PCA), the first principal component (PC1) explained 26.3% of the variation, and the second principal component (PC2) explained 17.43% of the variation (Figure 2A). The samples were separated by cultivars along PC2. Correlation analysis explained the strong correlation of sample repetition (Figure 2B).

The number of DEGs in two *E. ulmoides* varieties at three AR development stages were 6571 in H8S1 vs. H6S1, 6251 in H8S2 vs. H6S2, and 3538 in H8S3 vs. H6S3, with the highest number of DEGs discovered in the S1 stage (Figure 2C). In each stage, the number of up-regulated DEGs was greater than the number of down-regulated DEGs. DEGs were used for KEGG enrichment analysis, and several pathways that may play key roles in AR development were identified. The top 20 enriched metabolic pathways in each stage were displayed (Figure 2D–F). In H8S1 vs. H6S1, metabolic pathways such as phenylpropanoid biosynthesis, starch and sucrose metabolism, glycolysis/gluconeogenesis, flavonoid biosynthesis, and the pentose phosphate pathway were enriched (Figure 2D). In H8S2 vs. H6S2, metabolic pathways such as ribosome, phenylpropanoid biosynthesis, glycolysis/gluconeogenesis, ABC transporters, and cysteine and methionine metabolisms were enriched (Figure 2E). In H8S3 vs. H6S3, metabolic pathways such as plant–pathogen interaction, phenylpropanoid biosynthesis, ribosome, MAPK signaling pathway-plant, and starch and sucrose metabolisms were enriched (Figure 2F). Furthermore, phenylpropanoid biosynthesis and flavonoid biosynthesis were enriched in the S1, S2, and S3 stages, and isoflavonoid biosynthesis was enriched in both the S1 and S2 stages.

### 2.3. Construction and Analysis of the WGCNA

**Figure 3:**
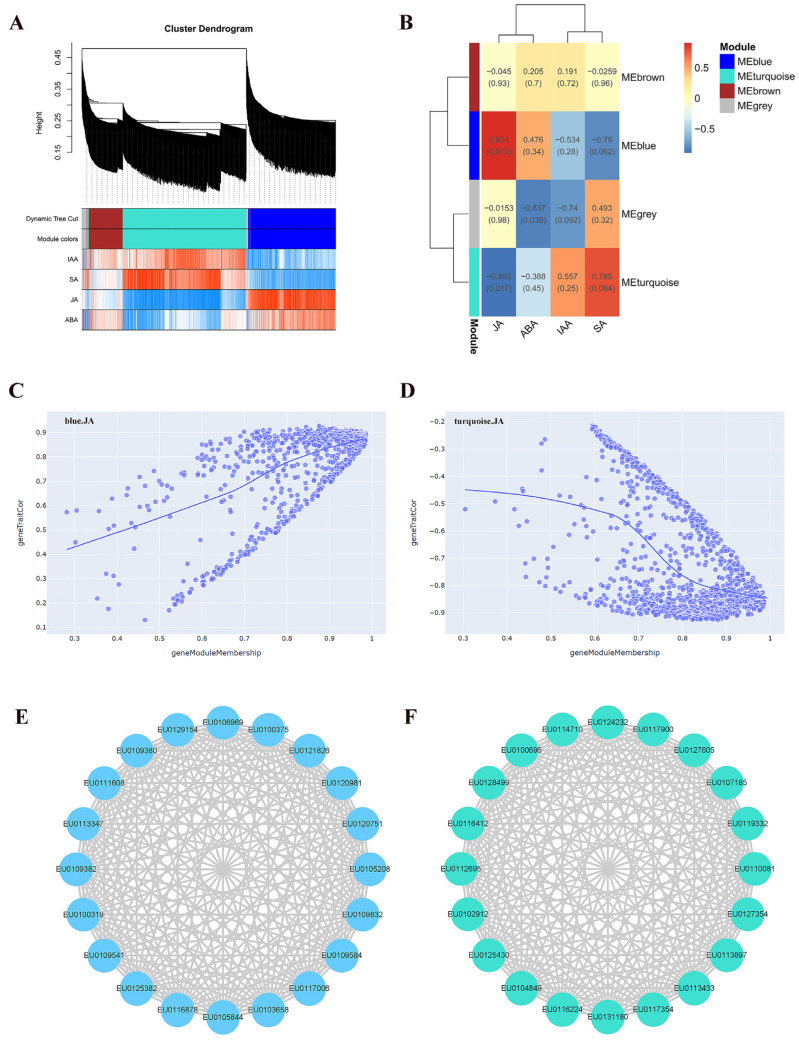
WGCNA of expressed genes with FPKM > 1 during *E. ulmoides* H6 AR development. (**A**) Hierarchical clustering tree depicting four co-expressed gene modules. (**B**) Heatmap of the correlation between modules and hormones. (**C**) Gene significance and module membership in MEblue-JA. (**D**) Gene significance and module membership in MEturquoise-JA. (**E**) Correlation network of 20 hub genes in the blue module. (**F**) Correlation network of 20 hub genes in the turquoise module.

**Figure 4:**
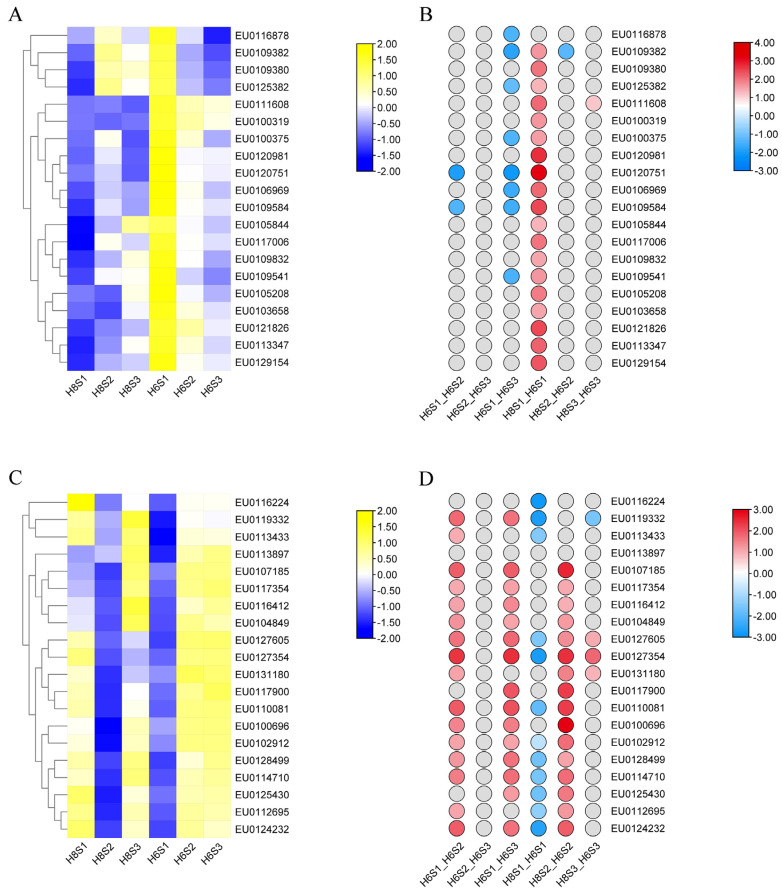
The differential expression of hub genes in the blue and turquoise modules significantly correlated with JA. (**A**) Expression levels of hub genes in the blue module. (**B**) Differential changes of hub genes in the blue module. (**C**) Expression levels of hub genes in the turquoise module. (**D**) Differential changes of hub genes in the turquoise module. The red circles, blue circles, and gray circles represent significant up-regulation, significant down-regulation, and no significant change for hub genes under corresponding comparison groups, respectively. The color depth of red circles and blue circles represents the size of log_2_ fold change.

All expressed genes were divided into four modules containing 685, 985, 270, and 60 genes in blue, turquoise, brown, and gray, respectively (Figure 3A). The genes in the blue module were positively correlated with JA and negatively correlated with SA, while the genes in the turquoise module were negatively correlated with JA and positively correlated with SA (Figure 3B). The significantly relevant genes were demonstrated by gene significance and module membership in MEblue-JA (Figure 3C) and MEturquoise-JA (Figure 3D). Twenty hub genes were identified in the blue and turquoise modules, respectively (Figure 3E,F). A total of 21 orthologous genes of these *E. ulmoides* hub genes were found in *A. thaliana* (Table S2). Compared with H8, 19 hub genes in the blue module were significantly up-regulated in the S1 stage in H6 (Figure 4A,B). The eight hub genes in the blue module were differentially expressed in three stages of AR development of H6, namely *EU0109382*, *EU0106969*, *EU0100375*, *EU0120751*, *EU0109584*, *EU0116878*, *EU0125382*, and *EU0109541* (Figure 4A,B). The results of the functional annotation of proteins exposed that EU0120751 was an F-box protein and that EU0125382 was a SAUR-like auxin-responsive family protein. Compared with H8, 19 hub genes in the turquoise module were differentially expressed in H6, most of which were significantly down-regulated in the S1 stage and up-regulated in the S2 stage (Figure 4C,D). A total of 18 hub genes in the turquoise module were differentially expressed, containing *EU0112695*, *EU0116412*, *EU0128499*, *EU0100696*, *EU0114710*, *EU0124232*, *EU0117900*, *EU0127605*, *EU0107185*, *EU0119332*, *EU0127354*, *EU0110081*, *EU0113433*, *EU0117354*, *EU0131180*, *EU0104849*, *EU0125430*, and *EU0102912* (Figure 4C,D). The results of the functional annotation of proteins indicated that EU0124232 belonged to the LOB protein and EU0128499 belonged to the B3 domain-containing AP2/ERF family transcription factor.

### 2.4. Metabolome Profile of H8 and H6 during AR Development

**Figure 5:**
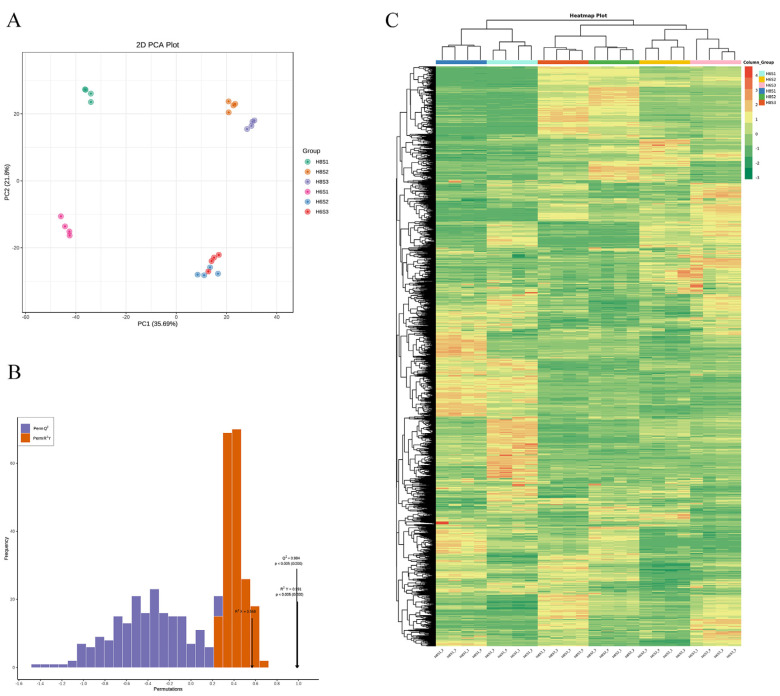
PCA, OPLS-DA, and expression profiling of 24 metabolome samples in three AR development stages: (**A**) PCA; (**B**) OPLS-DA; (**C**) expression profiling of all metabolites.

Based on the LC-QTOF platform, qualitative and quantitative analyses of the metabolome were conducted on 24 samples, resulting in the detection of 13,088 peaks, with the annotation of 2396 metabolites. PCA results indicated that the metabolites in these samples exhibited obvious differences, consistent with the AR phenotype and hormone levels (Figure 5A). The PC1 can explain 35.69% of the features of the original data set. According to PC1, the separation of the same *E. ulmoides* variety at different times (S1 stage and other two stages) was uncovered, indicating that the metabolites in the S2 and S3 stages were quite different from those in the S1 stage. Conspicuously, the treatments of different *E. ulmoides* varieties were separated on the PC2, and PC2 explained 21.8% of the characteristics of the original data set. Furthermore, OPLS-DA results indicated Q^2^ > 0.9, suggesting a highly reliable model (Figure 5B).

The differences in accumulation patterns of metabolites in different varieties and stages were manifested (Figure 5C). All metabolites were divided into six clusters. The content of metabolites in cluster 1 was the highest in H8S2 and H8S3, and the content in other groups was low. The content of metabolites in cluster 2 was the highest in H6S2 and H6S3 but lower in other groups. The content of metabolites in cluster 3 was the highest in H8S1 and H6S1 groups but lower in other groups. Different biological repeats were also clustered into a cluster, which indicated the good homogeneity of biological repeats and the high reliability of metabolic data. Above all, cluster results and PCA results indicated significant differences of metabolites in different *E. ulmoides* varieties and AR development stages.

**Figure 6:**
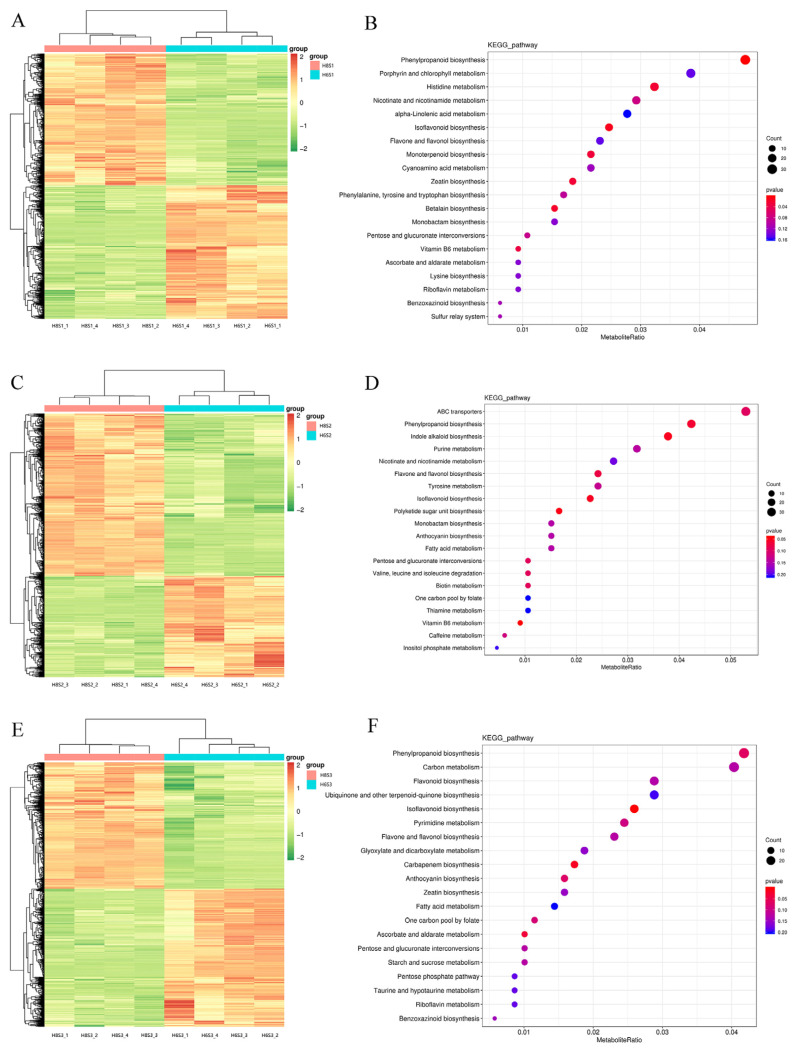
The DEMs and KEGG enrichment analyses during AR development. (**A**) DEMs in H6S1 vs. H8S1. (**B**) KEGG enrichment in H6S1 vs. H8S1. (**C**) DEMs in H6S2 vs. H8S2. (**D**) KEGG enrichment in H6S2 vs. H8S2. (**E**) DEMs in H6S3 vs. H8S3. (**F**) KEGG enrichment in H6S3 vs. H8S3.

A large number of DEMs were revealed in different comparison groups. In the comparison group of H6S1 vs. H8S1, 1361 DEMs were identified, of which 686 were up-regulated and 675 were down-regulated (Figure 6A). These DEMs were mainly enriched in phenylpropanoid biosynthesis, porphyrin and chlorophyll metabolism, histidine metabolism, nicotinate and nicotinamide metabolism, and alpha-linolenic acid metabolism pathways (Figure 6B). In the comparison group of H6S2 vs. H8S2, 1395 DEMs were exposed, of which 536 were up-regulated and 859 were down-regulated (Figure 6C). These DEMs were mainly enriched in ABC transporters, phenylpropanoid biosynthesis, indole alkaloid biosynthesis, purine metabolism, and nicotinate and nicotinamide metabolism (Figure 6D). In the comparison group of H6S3 vs. H8S3, 1402 DEMs were unveiled, of which 730 were up-regulated and 672 were down-regulated (Figure 6E). These DEMs were mainly enriched in phenylpropanoid biosynthesis, carbon metabolism, flavonoid biosynthesis, ubiquinone and other terpenoid-quinone biosynthesis, and isoflavonoid biosynthesis (Figure 6F). Phenylpropanoid biosynthesis was enriched in every stage of AR development.

### 2.5. Transcriptome and Metabolome of Key Pathways duing AR Development

**Figure 7:**
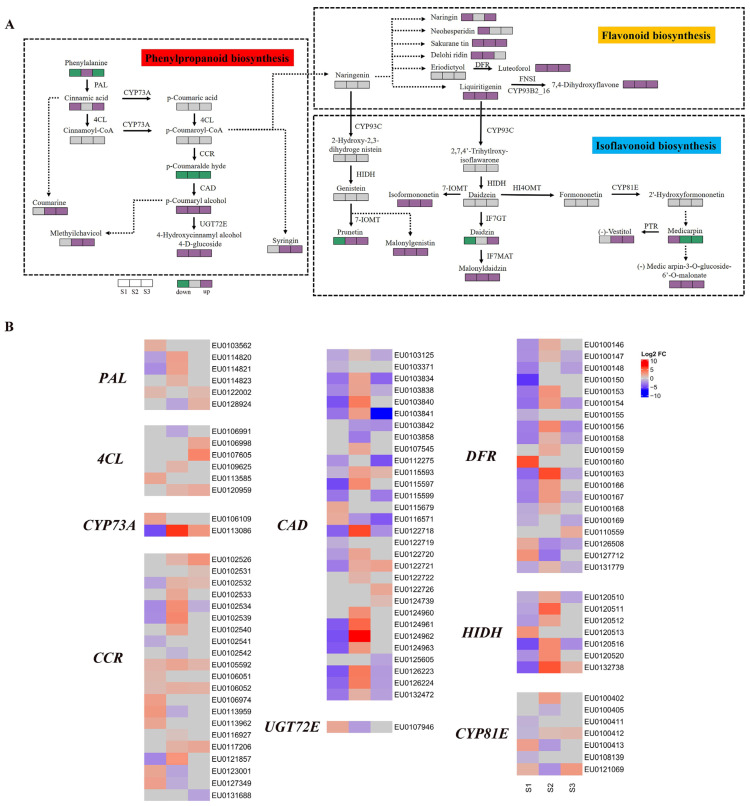
The differential expression profile of transcriptomics and metabolics in phenylpropanoid biosynthesis, flavonoid biosynthesis, and isoflavonoid biosynthesis in three stages during *E. ulmoides* AR development. (**A**) Metabolic pathway map and differential expression profile of metabolite contents. The purple boxes, green boxes, and gray boxes represent significant up-regulation, significant down-regulation, and no significant change for metabolite contents in H6S1 vs. H8S1, H6S2 vs. H8S2, and H6S3 vs. H8S3, respectively. (**B**) The differential expression profile of genes in the pathways. The red boxes, blue boxes, and gray boxes represent significant up-regulation, significant down-regulation, and no significant change for gene expression in H6S1 vs. H8S1, H6S2 vs. H8S2, and H6S3 vs. H8S3, respectively. The color depth of boxes represents the size of log_2_ fold change.

The key metabolic pathways in the AR development of *E. ulmoides* were constructed, including phenylpropanoid biosynthesis, flavonoid biosynthesis, and isoflavonoid biosynthesis. Functional genes and key metabolites involved in three pathways were identified. The difference profiles of gene expression and metabolite content between two *E. ulmoides* cultivars were exhibited in H8S1 vs. H6S1, H8S2 vs. H6S2, and H8S3 vs. H6S3 (Figure 7).

In the phenylpropanoid biosynthesis pathway, compared with H8, the metabolite content of H6 showed significant differences in three stages of AR development. The metabolite content of *p*-coumaryl alcohol and 4-hydroxycinnamyl alcohol 4-D-glucoside of H6 was significantly up-regulated in all three stages. The metabolite content of the coumarine, mlethyilchavicol, and syringin of H6 was significantly up-regulated at the S2 stage and the S3 stage. In addition, the content of some metabolites of H6 was also up-regulated or down-regulated in different stages, including phenylalanine, cinnamic acid, and *p*-coumaralde hyde (Figure 7A). The DEG profiles uncovered that six phenylalanine ammonialyase (PAL), six 4-coumarate: CoA ligase (4CL), two cytochrome P450 73A (CYP73A), 21 cinnamoyl-CoA reductase (CCR), 30 cinnamyl-alcohol dehydrogenase (CAD), and one coniferyl-alcohol glucosyltransferase (UGT72E) genes were up-regulated or down-regulated in three stages of AR development. In the S2 and S3 stages, more than half of the CCR genes were significantly up-regulated. The two CCR genes (EU0105592 and EU0106052) were significantly up-regulated in three stages. Most CAD genes were significantly up-regulated in the S2 stage and down-regulated in the S1 and S3 stages. As the up-regulated DEGs, EU0113086 (CYP73A), EU0122718 (CAD), and EU0124962 (CAD) had the largest change folds, which were 6.3, 5.8, and 7.0 respectively (Figure 7B).

In the flavonoid biosynthesis pathway, compared with H8, the metabolite content of H6 also had significant differences. The metabolite content of sakurane tin, luteoforol, liquiritigenin, and 7,4-dihydroxyflavone of H6 was significantly up-regulated in all three stages. In addition, the metabolite content of H6, such as naringin, neoheperidin, and delohiridin, also suggested significant up-regulation (Figure 7A). A total of 20 bifunctional dihydroflavonol 4-reductase (DFR) genes were differentially expressed during AR development. The most DFR genes were up-regulated at S2 stage, and the most DFR genes were down-regulated at S1 and S3 stages. EU0100163 (DFR) demonstrated the largest change fold in the S2 stage (Figure 7B).

In the isoflavonoid biosynthesis pathway, significant differences in metabolite content and gene expression between H6 and H8 were also observed. The content of four metabolites of H6 were significantly up-regulated in all three stages, including malonylgenistin, isoformononetin, malonyldaidzin, and (-) Medic arpin-3-O-glucoside-6′-O-malonate (Figure 7A). The seven 2-hydroxyisoflavanone dehydratase (HIDH) genes were differentially expressed during AR development. The vast majority of HIDH genes conveyed the same expression pattern in the S1 and S2 stages, i.e., down-regulated expression in the S1 stage and up-regulated expression in the S2 stage. EU0132738 (HIDH) and EU0120511 (HIDH) showed the largest change fold in the S2 stage. Additionally, cytochrome P450 81E (CYP8IE) genes were also differentially expressed in different stages of AR development (Figure 7B).

### 2.6. The Cis-Regulation of Hub Genes by lncRNAs

A total of 5862 candidate lncRNAs were screened (Figure S1A), containing 4500 lincRNAs (76.8%), 469 antisense lncRNAs (8%), 609 intronic lncRNAs (10.4%), and 284 sense lncRNAs (4.8%) (Figure S1B). Some basic characteristics of lncRNA and mRNA were compared (Figure S1C–F). 

**Figure 8:**
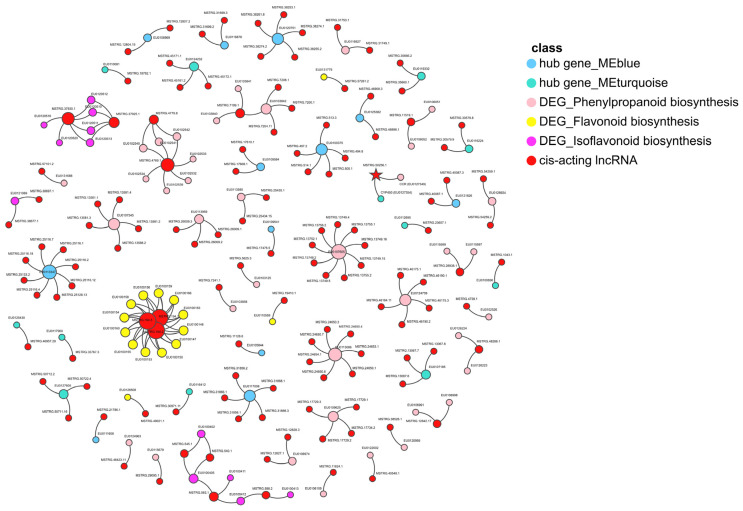
The 130 lncRNA cis-target hub genes in modules that significantly correlated with JA and DEGs in phenylpropanoid biosynthesis, flavonoid biosynthesis, and isoflavonoid biosynthesis pathway. Each type of factor is marked with various colors. The lncRNA MSTRG.50256.1 is marked with a pentagram.

The 130 lncRNAs with potential regulatory capability were screened, and cis-targeting hub genes in the modules significantly were correlated with JA and DEGs in phenylpropanoid biosynthesis, flavonoid biosynthesis, and isoflavonoid biosynthesis pathways (Figure 8). Altogether, 17 lncRNAs exhibited differential expressions, and 113 lncRNAs possessed potential regulatory effects during the AR development of *E. ulmoides* (Figure S2). Eminently, MSTRG.50256.1 was the cis-acting lncRNA of the cytochrome P450 (CYP450) superfamily gene (EU0127354) in the module, which significantly correlated with JA, and it performed simultaneously as the cis-acting lncRNA of the CCR gene (EU0127349) in the phenylpropanoid biosynthesis pathway (Figure 8).

## 3. Discussion

AR formation is the crucial process of cutting propagation and the primary bottleneck restricting the survival of cuttings for horticultural and forestry plants [49,50]. Therefore, understanding the regulation of AR formation is extremely necessary in cultivation and breeding. Complex interactions among genotypes, hormone crosstalk, metabolic states, epigenetic regulation, and other factors are considered to shape the AR characteristics of woody plants [51]. In this study, the key molecules and important pathways of *E. ulmoide* AR formation were comprehensively analyzed by integrating physiological data and whole transcriptomes and metabolomes. What needs to be emphasized is that these results only revealed preliminary associations or patterns between the key molecules and important pathways and AR development. These potential causal relationships provide rich genetic and metabolite resources for future research, and the determination of causal relationships needs to be explored from multiple perspectives in subsequent work. This study significantly promotes the comprehension of the regulation of AR formation in *E. ulmoides* and other plants.

### 3.1. Differences in Plant Hormone Content between Two E. ulmoides Cultivars

The IAA level of H6 (easy-to-root cultivar) was significantly higher than that of H8 (difficult-to-root cultivar), which indicated that IAA is an important endogenous hormone that regulates AR formation. The level of IAA increased during the critical period of AR formation, significantly promoting the occurrence of AR. Auxin is a key hormone affecting the formation of AR in plants, and it can interact with other hormones to jointly affect the formation of AR [13]. The level of IAA can be used as one of the important indexes to judge the ability of cutting the roots of improved varieties of *E. ulmoides.*

Notably, the JA level of two *E. ulmoides* cultivars decreased significantly from the 0th day (S1 stage) to the 18th day (S2 stage). The JA level of petunia, carnation, and pea cuttings reached the highest level within a period of time after cutting and then decreased [52,53,54]. JA is a hormone involved in abiotic stress response [55,56,57,58]. The higher JA level in the 0th day and the lower JA level in the 18th day of H6 variety may be important factors for superior AR formation ability. The dual effects of JA on AR development may be related to level and stage. This view is supported by some studies [10]. For example, the lower wound-induced JA level of transgenic plants reduced the numbers of AR in petunia [15]. At the same time, the SA level also changed reversely in the two varieties from the 0th day to the 18th day with a different order than the JA level, which implies that there may be distinct regulatory mechanisms and interfering signal transduction between JA and SA during the early stages of AR formation [59,60,61].

### 3.2. Several Hub Genes Regulate AR Development

JA can interact with various hormones to regulate AR formation [10,62]. In this study, several hub genes that can directly or indirectly regulate hormone levels were found in the gene module and were significantly correlated with JA. The gene modules with positive correlation and negative correlation of JA content were manifested by WGCNA. Twenty hub genes were screened from the two modules, and most of them were differentially expressed in different stages of AR development in *E. ulmoides*. *EU0120751* and *EU0125382* were hub genes in modules with significant positive correlation with the JA level. EU0120751 was an F-box protein. In *A. thaliana*, TIR1 and auxin-signaling F-box 2 protein could modulate JA homeostasis and control AR initiation by specific sensing complexes with IAA6, IAA9, and/or IAA17 [63]. EU0125382 was a SAUR-like auxin-responsive family protein. The WOX11-SAUR-auxin signaling regulatory module was essential for AR development in poplar [64]. *EU0120751* and *EU0125382* may directly or indirectly affect the accumulation of JA and control the formation of AR of *E. ulmoides*. *EU0124232* and *EU0128499* were hub genes in modules with a significantly negative correlation with JA level. EU0124232 belonged to the LOB protein. In apple, the *MdLBDs* promoter contained many cis-acting regulatory elements related to the response of growth regulators, and the over-expression of *MdLBD16a* in tomato increased the number of AR [65]. Our study revealed that *AT5G06080* was the orthologous gene of *EU0124232*. It has been demonstrated that *AtLBD33* (*AT5G06080*) played an important regulatory role in *A. thaliana* root development [66,67]. EU0128499 belonged to the B3 domain-containing AP2/ERF family transcription factor. AP2/ERF protein played a vital role in signal pathways mediated by different plant hormones [68]. *OsAP2*/*ERF-40* could promote AR development in rice [69]. *EU0120751*, *EU0125382*, *EU0124232*, and *EU0128499* may modulate the AR development of *E. ulmoides* by regulating the plant hormone network composed of JA, IAA, ethylene, and other hormones, which needs to be further studied by molecular biology methods, such as CRISPR, in the future [70]. Previous studies initially defined the AR development phase of *E. ulmoides* [8], and the functional mechanism of each key gene at every developmental stage can be scrutinized in prospective research endeavors.

### 3.3. Metabolic Pathway Enrichment of DEGs and DEMs during AR Development

The integrated analysis of transcriptomics and metabolomics revealed that DEGs and DEMs were co-enriched in phenylpropanoid biosynthesis, flavonoid biosynthesis, and isoflavonoid biosynthesis, suggesting that these three metabolic pathways were essential to form AR for *E. ulmoides*. The phenylpropanoid biosynthesis pathway was also enriched during AR formation in other plants [42,71,72]. Many genes were differentially expressed, including six *PAL*, six *4CL*, two *CYP73A*, 21 *CCR*, 30 *CAD*, one *UGT72E*, 20 *DFR*, seven *HIDH*, and seven *CYP8IE*. The *PAL* genes have been demonstrated to affect the formation of AR in walnut, wheat, and *Echinacea purpurea* [73,74,75]. Conspicuously, the genes of *E. ulmoides* H6 were generally significantly up-regulated. Therefore, the difference in gene expression level on the 18th day may be an important reason for the difference in AR development between the two *E. ulmoides* varieties. Some genes with the same expression pattern were observed, such as *CAD* genes (*EU0103834*, *EU0103838*, and *EU0122718*). At the same time, genes with opposite expression patterns also were found, such as *CCR* genes (*EU0121857* and *EU0123001*). This suggests that these genes may have manifold effects in AR formation.

### 3.4. LncRNAs Regulate Plant Hormone Signal Transduction during AR Development

LncRNAs can regulate mRNAs by co-localization [76,77,78], and they exhibit a critical regulatory role for AR formation in forest trees and other plants [79,80,81]. For example, the lncWOX11a suppressed AR development by regulating the expression of *PeWOX11a* in poplar [82]. The lncWOX5 negatively regulated *WOX5*, and lncWOX11 positively regulated *WOX11* during the AR formation of poplar [46]. The overexpression of lncWOX5 negatively regulated the development of AR in *Populus* [83]. A total of 130 cis-acting lncRNAs were identified associated with the hub genes of modules significantly correlated with JA and DEGs in the phenylpropanoid biosynthesis, flavonoid biosynthesis, and isoflavonoid biosynthesis pathway, with 17 of them exhibiting significant differential expression during root cuttings in favorable conditions of other growth environment factors in *E. ulmoides*. 

MSTRG.50256.1, as an exemplar of cis-acting lncRNA, potentially regulates the *CYP450* superfamily gene (*EU0127354*) in modules significantly correlated with JA and the *CCR* family gene (*EU0127349*) in the phenylpropanoid biosynthesis pathway. CYP450 is a huge enzyme superfamily with heme as a cofactor, which is known as a universal biocatalyst and performs an important feature in the metabolic process associated with plant growth and development, such as fatty acids, phytosterols, plant hormones, and phenylpropanoids [84,85,86,87]. In the phenylpropanoid biosynthesis pathway, CYP450 monooxygenase 98 catalyzed a rate-limiting step [88]. In soybean, *CYP728H1* was proven to be co-expressed with the genes related to the phenylpropanoid metabolism [89]. In *Linum usitatissimum* L., seven gene families, namely *CYP73*, *74*, *75*, *76*, *77*, *84*, and *709*, encoded enzymes associated with the phenylpropanoid metabolism [90]. AR can be formed in many situations, including abiotic stresses and exogenous hormones, in addition to cuttings [91,92]. When cuttings are accompanied by floods and exogenous hormones, the functions of these lncRNAs may be stimulated. MSTRG.50256.1, as the cis-acting lncRNA of the *CYP450* gene in the module significantly correlated with JA and the *CCR* gene in the phenylpropanoid biosynthesis pathway, emphasizes a global regulatory function that needs to be revealed urgently in the future during the AR development of *E. ulmoides*.

## 4. Materials and Methods

### 4.1. Plant Materials and Sampling

**Figure 9:**
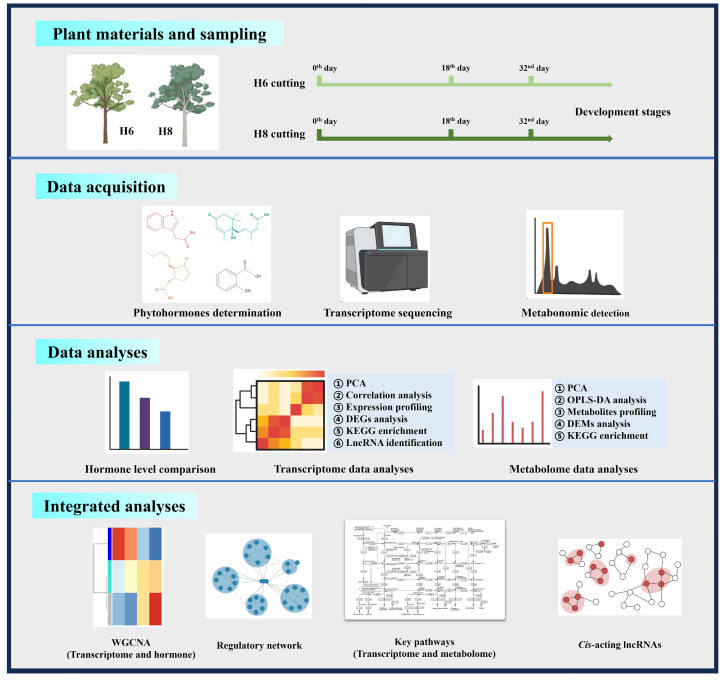
An overall framework of materials and methods. The drawing was created with BioRender (https://www.biorender.com/ (accessed on 24 December 2023)).

The semi-lignified cuttings were taken from three-year-old *E. ulmoides* H6 and *E. ulmoides* H8, cultivated from the *Eucommia* Engineering Research Center of State Forestry and Grassland experimental base (34°500′ N, 112°330′ E, altitude: 108 m) located in the Mengzhou, Henan province, China (Figure 9). The semi-lignified spring shoots, measuring 10–15 cm in length, were chosen as cuttings, and 2–3 mature leaves on the top tender shoots were retained. The cuttings were broken manually from the base without scissors. These cuttings were dipped in the special rooting agent (patent number: CN202011236585.9) for 10 s, and they were inserted into the cutting bed that was disinfected with a 500-fold dilution of 40% carbendazim wettable powder before cutting. The cutting bed was 8 m long and 1.5 m wide, laid with 0.15 m of coarse river sand at the bottom, and covered with 0.15 m of matrix (turfy soil:perlite = 3:1) at the top. The ambient temperature was controlled at 20~29 °C, and the relative humidity was controlled above 90%. *E. ulmoides* cuttings were studied after 0, 18, and 32 days, which were recorded as S1, S2, and S3 respectively. These sampling times were considered by referring to our previous study on the primary definition of the AR development phase of *E. ulmoides* [8]. Additionally, for convenience, S1–S3 stages of H6 and H8 cuttings were named H6S1-H6S3 and H8S1-H8S3. Stem segments with lengths of 1–3 cm at the base of cuttings were collected, and two biological replicates were made for whole transcriptome strand-specific RNA sequencing and four biological replicates for LC-MS/MS non-targeted determination, respectively. All samples were immediately frozen in liquid nitrogen and stored at −80 °C.

### 4.2. Determination of the Content of Plant Endogenous Hormones

High performance liquid chromatography–tandem mass spectrometry (HPLC–MS/MS) was used to determine the content of endogenous hormones, including IAA, ABA, JA, and SA. The determination conditions were as follows: BEH C18 column (2.1 × 100 mm, 1.8 µm), 0.1% formic acid aqueous solution as mobile phase A, acetonitrile as mobile phase B, linear gradient elution, flow rate 0.25 mL/min, injection volume 5 µL, and column temperature 40 °C. The mass spectrometry parameters were as follows. Electrospray ionization source (ESI) was in positive and negative ion ionization mode. The ion source temperature was 500 °C. The ion source voltage was 5500–4500 V. The curtain gas was 30 psi. The atomization gas and auxiliary gas were both 50 psi. Multiple Reaction Monitoring (MRM) was adopted for scanning. Comparisons were analyzed using the one-way analysis of variance (ANOVA) test.

### 4.3. RNA Extraction, Library Construction and Sequencing

Total RNA was extracted using TRIzol (Invitrogen, Waltham, MA, USA) following the manufacturer’s protocol. RNA quality was determined by 1% agarose gel electrophoresis. The concentration and purity were measured using Nanodrop 2000 (Thermo Fisher Scientific, Bothell, WA, USA). RNA integrity was checked by Agient 2100 (Agilent Technologies, Santa Clara, CA, USA). rRNA was removed using the Epicentere Ribo-zero™ rRNA Removal Kit (Epicentere, Middletown, DE, USA). Sequencing libraries were prepared by using the NEBNextR Ultra™ Directional RNA Library Prep Kit for Illumina^®^ (NEB, San Diego, CA, USA) following the manufacturer’s instructions. Finally, 12 libraries were constructed and sequencing was performed on an Illumina NovaSeq 6000 platform, and 150-bp paired-end reads were obtained.

### 4.4. Transcriptome Assembly, lncRNA Identification, and Differential Expression Analysis

Clean reads were obtained by removing reads containing adapter, poly-N, and low-quality reads from raw data. At the same time, the Q20, Q30, GC-content, and sequence duplication level of the clean data were calculated. All downstream analyses were based on clean data with high quality. The clean reads were mapped to the *E. ulmoides* genome [48] using HISAT2 v2.0.4 [93]. Then, the transcriptome was assembled by StringTie v1.3.1 [94] based on the reads mapped to the reference genome. The gffcompare program was used to annotate the assembled transcripts.

The unknown transcripts were used to screen for putative lncRNAs. Transcripts with a length of ≥200 bp, number of exons ≥ 2, and expression levels of fragments per kilobase of exons per million fragments mapped (FPKM) ≥ 0.1 were selected as lncRNA candidates and further screened using four computational approaches (CPC/CNCI/Pfam/CPAT) that could distinguish protein-coding genes from non-coding genes. Using Perl script, the neighboring genes within 100 kb upstream and downstream of lncRNAs were used as cis-target genes of lncRNAs. The network diagram of the relationship between cis-acting lncRNAs and genes was shown by ChiPlot (https://www.chiplot.online/ (accessed on 20 October 2023)). 

StringTie v1.3.1 was used to calculate the FPKM of both lncRNAs and coding genes in each sample. The PCA of transcriptome samples was performed using the Metware Clouds (https://cloud.metware.cn (accessed on 10 July 2023)). Differential expression analysis of two groups was performed using the DESeq R package [95]. The DEGs and differently expressed cis-acting lncRNAs (DELs) were identified from six combinations of the H6S1 vs. H6S2, H6S2 vs. H6S3, H6S1 vs. H6S3, H6S1 vs. H8S1, H6S2 vs. H8S2, and H6S3 vs. H8S3 groups. The criteria were |log_2_ fold change| ≥ 1 and *p*-value ≤ 0.01. The expression level (FPKM) and change fold of DEGs and DELs were shown by TBtools [96]. The KEGG enrichment analyses of DEGs were carried out using the clusterProfiler v3.14.3 R package [97].

### 4.5. WGCNA of Hormone Traits and All Expressed Genes

WGCNA was performed using hormone level traits of IAA, ABA, JA, and SA and all genes with FPKM > 1 of *E. ulmoides* H6 using ImageGP (https://www.bic.ac.cn/ImageGP/ (accessed on 20 September 2023)) [98]. The protein functional annotation of hub genes was performed by the Conserved Domain Database (https://www.ncbi.nlm.nih.gov/cdd (accessed on 6 October 2023)) [99]. The protein–protein interaction networks of hub genes were drawn using Cytoscape v3.8.2 [100]. The longest transcript was selected to represent each gene with multiple isoforms. Protein sequences of *E. ulmoides* and *A. thaliana* were used for family classification using OrthoFinder v2.5.4 [101].

### 4.6. Metabolite Profiling and Data Analysis

Lyophilized powder (50 mg) was weighed and extracted using 1 mL of extract solution (methanol: acetonitrile: water = 2:2:1) and was vortexed for 30 s. It was homogenized in ball mill for 10 min at 45 Hz, then put in an ultrasonic 10 min ice-water bath, and incubated at −20 °C for 1 h. The extracts were centrifuged at 12,000 rpm for 15 min at 4 °C, and supernatant (500 μL) was transferred into EP tubes, then dried in a vacuum concentrator, and 160 μL of extraction solvent (acetonitrile: water= 1:1) was added for reconstitution. It was vortexed for 30 s and sonicated for 10 min (ice-water bath). Finally, the supernatant was obtained by centrifugation at 4 °C at 12,000 rpm for 15 min and transferred to a 2 mL glass vial through a 0.22 µm membrane. The filtrate was analyzed using a UPLC–MS system (UPLC: Acquity I-Class PLUS, Waters, Milford, MA, USA; MS: Xevo G2-XS QTOF, Waters, Milford, MA, USA). The ESI source conditions were set as follows: capillary voltage: 2500 V (positive ion mode) or −2000 V (negative ion mode); cone voltage: 30 V; ion source temperature: 150 °C; desolvent gas temperature 500 °C; backflush gas flow rate: 50 L/h; desolventizing gas flow rate: 800 L/h.

Metabolites were characterized based on secondary spectral information using the METLIN and MVDBV database. They were quantified by triple quadruple mass spectrometry in the multiple reaction monitoring mode. PCA analysis, OPLS-DA analysis, and expression profiling of all metabolites of 24 metabolome samples were performed using the Metware Clouds (https://cloud.metware.cn (accessed on 10 July 2023)). The differently expressed metabolites (DEMs) were obtained from 3 combinations of H6S1 vs. H8S1, H6S2 vs. H8S2, and H6S3 vs. H8S3 groups. The criteria were the threshold of variable importance in projection ≥ 1, |log_2_ fold change| ≥ 1, and *p*-value ≤ 0.05. The KEGG enrichment analyses of DEMs were carried out using clusterProfiler v3.14.3 R package.

## 5. Conclusions

In this study, the pivotal factors and main pathways related to the AR formation of *E. ulmoides* were identified by comparing the physiological indexes, transcriptomes, and metabolomes of two *E. ulmoides* varieties with different AR formation abilities. Significant differences of IAA, ABA, JA, and SA levels between two varieties were found. The hub genes significantly related to JA were demonstrated, such as *F-box*, *SAUR-like*, *LOB*, and *AP2/ERF*. DEGs and DEMs were enriched in multiple pathways, including phenylpropanoid biosynthesis, flavonoid biosynthesis, and isoflavonoid biosynthesis. Most *PAL*, *CCR*, *CAD*, *DFR*, and *HIDH* genes were significantly up-regulated on the 18th day in easy-to-root cultivar. The 130 cis-targeting lncRNAs with potential regulatory roles in AR formation were revealed. The role of these key molecules and pathways in AR development can be further researched through CRISPR, single-cell, and spatial omics technologies in future studies. In conclusion, these results provide an important theoretical foundation for understanding the molecular mechanism of AR formation of *E. ulmoides*.

## Data Availability

Data are contained within the article or Supplementary Materials.

## Supplementary Materials

The following supporting information can be downloaded at: https://www.mdpi.com/article/10.3390/plants13010136/s1, Figure S1: The basic characteristics of lncRNA and mRNA during *E. ulmoides* AR development; Figure S2: The differential expression of 130 cis-acting lncRNAs during cutting rooting in favorable growth environment; Table S1: Summary of strand-specific RNA sequencing data; Table S2: *E. ulmoides* hub genes in blue module and turquoise module and *A. thaliana* orthologous genes.

## References

[1] Dong C.B., Zhang Z.Y., Shao Q.Y., Yao T., Hu H.Y., Huang J.Z., Liang Z.Q., Han Y.F. (2022). Deciphering the effects of genetic characteristics and environmental factors on pharmacological active ingredients of *Eucommia ulmoides*. Ind. Crops Prod..

[2] Liu X., Wang X.Z., Kang K., Sun G.T., Zhu M.Q. (2022). Review on extraction, characteristic, and engineering of the *Eucommia ulmodies* rubber for industrial application. Ind. Crops Prod..

[3] Wang C.C., Gong H.M., Feng M., Tian C.L. (2023). Phenotypic Variation in Leaf, Fruit and Seed Traits in Natural Populations of *Eucommia ulmoides*, a Relict Chinese Endemic Tree. Forests.

[4] He X.R., Wang J.H., Li M.X., Hao D.J., Yang Y., Zhang C.L., He R., Tao R. (2014). *Eucommia ulmoides* Oliv.: Ethnopharmacology, phytochemistry and pharmacology of an important traditional Chinese medicine. J. Ethnopharmacol..

[5] Ruan L., Lu L., Zhao X.Y., Xiong W.W., Xu H.Y., Wu S.Z. (2022). Effects of natural antioxidants on the oxidative stability of *Eucommia ulmoides* seed oil: Experimental and molecular simulation investigations. Food Chem..

[6] Wei X.N., Peng P., Peng F., Dong J.E. (2021). Natural Polymer *Eucommia ulmoides* Rubber: A Novel Material. J. Agric. Food Chem..

[7] Wendling I., Trueman S.J., Xavier A. (2014). Maturation and related aspects in clonal forestry—Part II: Reinvigoration, rejuvenation and juvenility maintenance. New Forest..

[8] Lv G.X., Qing J., Du H.Y., Du Q.X., Meng Y.D., He F., Liu P.F., Du L.Y., Wang L. (2021). Comparing Rooting Ability and Physiological Changes of Two *Eucommia ulmoides* Improved Varieties. Forests.

[9] Brunoni F., Vielba J.M., Sánchez C. (2022). Plant Growth Regulators in Tree Rooting. Plants.

[10] Druege U., Hilo A., Pérez-Pérez J.M., Klopotek Y., Acosta M., Shahinnia F., Zerche S., Franken P., Hajirezaei M.R. (2019). Molecular and physiological control of adventitious rooting in cuttings: Phytohormone action meets resource allocation. Ann. Bot..

[11] Li S.W. (2021). Molecular Bases for the Regulation of Adventitious Root Generation in Plants. Front. Plant Sci..

[12] Lakehal A., Bellini C. (2019). Control of adventitious root formation: Insights into synergistic and antagonistic hormonal interactions. Physiol. Plant..

[13] Pacurar D.I., Perrone I., Bellini C. (2014). Auxin is a central player in the hormone cross-talks that control adventitious rooting. Physiol. Plant..

[14] Cai X.T., Xu P., Zhao P.X., Liu R., Yu L.H., Xiang C.B. (2014). *Arabidopsis ERF109* mediates cross-talk between jasmonic acid and auxin biosynthesis during lateral root formation. Nat. Commun..

[15] Lischweski S., Muchow A., Guthörl D., Hause B. (2015). Jasmonates act positively in adventitious root formation in petunia cuttings. BMC Plant Biol..

[16] Bernabé-Antonio A., Castro-Rubio C., Rodríguez-Anda R., Silva-Guzmán J.A., Manríquez-González R., Hurtado-Díaz I., Sánchez-Ramos M., Hinojosa-Ventura G., Romero-Estrada A. (2023). Jasmonic and Salicylic Acids Enhance Biomass, Total Phenolic Content, and Antioxidant Activity of Adventitious Roots of *Acmella radicans* (Jacq.) R.K. Jansen Cultured in Shake Flasks. Biomolecules.

[17] Fattorini L., Falasca G., Kevers C., Mainero Rocca L., Zadra C., Altamura M.M. (2009). Adventitious rooting is enhanced by methyl jasmonate in tobacco thin cell layers. Planta.

[18] Fattorini L., Veloccia A., Della Rovere F., D’Angeli S., Falasca G., Altamura M.M. (2017). Indole-3-butyric acid promotes adventitious rooting in *Arabidopsis thaliana* thin cell layers by conversion into indole-3-acetic acid and stimulation of anthranilate synthase activity. BMC Plant Biol..

[19] Fattorini L., Hause B., Gutierrez L., Veloccia A., Della Rovere F., Piacentini D., Falasca G., Altamura M.M. (2018). Jasmonate promotes auxin-induced adventitious rooting in dark-grown *Arabidopsis thaliana* seedlings and stem thin cell layers by a cross-talk with ethylene signalling and a modulation of xylogenesis. BMC Plant Biol..

[20] Chen K.M., Guo B., Yu C.M., Chen P., Chen J.K., Gao G., Wang X.F., Zhu A.G. (2021). Comparative Transcriptome Analysis Provides New Insights into the Molecular Regulatory Mechanism of Adventitious Root Formation in Ramie (*Boehmeria nivea* L.). Plants.

[21] Li S.W., Leng Y., Feng L., Zeng X.Y. (2014). Involvement of abscisic acid in regulating antioxidative defense systems and IAA-oxidase activity and improving adventitious rooting in mung bean *Vigna radiata* (L.) Wilczek seedlings under cadmium stress. Environ. Sci. Pollut. Res..

[22] Zeng Y.W., Verstraeten I., Trinh H.K., Heugebaert T., Stevens C.V., Garcia-Maquilon I., Rodriguez P.L., Vanneste S., Geelen D. (2021). *Arabidopsis* Hypocotyl Adventitious Root Formation Is Suppressed by ABA Signaling. Genes.

[23] Pasternak T., Groot E.P., Kazantsev F.V., Teale W., Omelyanchuk N., Kovrizhnykh V., Palme K., Mironova V.V. (2019). Salicylic Acid Affects Root Meristem Patterning via Auxin Distribution in a Concentration-Dependent Manner. Plant Physiol..

[24] Dong C.J., Liu X.Y., Xie L.L., Wang L.L., Shang Q.M. (2020). Salicylic acid regulates adventitious root formation via competitive inhibition of the auxin conjugation enzyme CsGH3.5 in cucumber hypocotyls. Planta.

[25] Liu W., Yu J., Ge Y.C., Qin P., Xu L. (2018). Pivotal role of *LBD16* in root and root-like organ initiation. Cell Mol. Life Sci..

[26] Ye B.B., Shang G.D., Pan Y., Xu Z.G., Zhou C.M., Mao Y.B., Bao N., Sun L.J., Xu T.D., Wang J.W. (2020). *AP2/ERF* Transcription Factors Integrate Age and Wound Signals for Root Regeneration. Plant Cell.

[27] Hu X.M., Xu L. (2016). Transcription Factors *WOX11/12* Directly Activate *WOX5/7* to Promote Root Primordia Initiation and Organogenesis. Plant Physiol..

[28] Gutierrez L., Mongelard G., Floková K., Pacurar D.I., Novák O., Staswick P., Kowalczyk M., Pacurar M., Demailly H., Geiss G. (2012). Auxin Controls *Arabidopsis* Adventitious Root Initiation by Regulating Jasmonic Acid Homeostasis. Plant Cell.

[29] Lee H.W., Cho C., Pandey S.K., Park Y., Kim M.J., Kim J. (2019). *LBD16* and *LBD18* acting downstream of *ARF7* and *ARF19* are involved in adventitious root formation in *Arabidopsis*. BMC Plant Biol..

[30] Trupiano D., Yordanov Y., Regan S., Meilan R., Tschaplinski T., Scippa G.S., Busov V. (2013). Identification, characterization of an *AP2/ERF* transcription factor that promotes adventitious, lateral root formation in *Populus*. Planta.

[31] Yin H.J., Li M.Z., Lv M.H., Hepworth S.R., Li D.D., Ma C.F., Li J., Wang S.M. (2020). *SAUR15* Promotes Lateral and Adventitious Root Development via Activating H^+^-ATPases and Auxin Biosynthesis. Plant Physiol..

[32] Wang H.M., Xie Y.H., Liu W.S., Tao G.Y., Sun C., Sun X.M., Zhang S.G. (2020). Transcription factor *LkWOX4* is involved in adventitious root development in *Larix kaempferi*. Gene.

[33] Zhang Y., Yang X.Q., Cao P., Xiao Z.A., Zhan C., Liu M.F., Nvsvrot T., Wang N. (2020). The *bZIP53*-IAA4 module inhibits adventitious root development in *Populus*. J. Exp. Bot..

[34] Chen X.D., Cheng J.F., Chen L.Q., Zhang G.F., Huang H., Zhang Y.J., Xu L. (2016). Auxin-Independent NAC Pathway Acts in Response to Explant-Specific Wounding and Promotes Root Tip Emergence during de Novo Root Organogenesis in *Arabidopsis*. Plant Physiol..

[35] Du X.L., Cao X., Yin C.R., Tang Z., Du W., Ban Y.Y., Cheng J.L. (2017). Comprehensive Analysis of *R2R3-MYB* Genes During Adventitious Root Formation in Cuttings of *Morus alba*. J. Plant Growth Regul..

[36] Wang P., Ma L.L., Wang S.A., Li L.F., Wang Q., Yang R.T., Li Y. (2019). Identification and Analysis of a Candidate *WRKY* Transcription Factor Gene Affecting Adventitious Root Formation Using Association Mapping in *Catalpa Scop*. DNA Cell Biol..

[37] Ai Y., Qian X., Wang X.Q., Chen Y.L., Zhang T.J., Chao Y.H., Zhao Y. (2023). Uncovering early transcriptional regulation during adventitious root formation in *Medicago sativa*. BMC Plant Biol..

[38] Gaillochet C., Lohmann J.U. (2015). The never-ending story: From pluripotency to plant developmental plasticity. Development.

[39] Vielba J.M., Rico S., Sevgin N., Castro-Camba R., Covelo P., Vidal N., Sánchez C. (2022). Transcriptomics Analysis Reveals a Putative Role for Hormone Signaling and MADS-Box Genes in Mature Chestnut Shoots Rooting Recalcitrance. Plants.

[40] Dong N.Q., Lin H.X. (2021). Contribution of phenylpropanoid metabolism to plant development and plant-environment interactions. J. Integr. Plant Biol..

[41] Sharma A., Shahzad B., Rehman A., Bhardwaj R., Landi M., Zheng B.S. (2019). Response of Phenylpropanoid Pathway and the Role of Polyphenols in Plants under Abiotic Stress. Molecules.

[42] Wang S.T., Sun G.D., Luo Y., Qian W.J., Fan K., Ding Z.T., Hu J.H. (2022). Role of IAA and Primary Metabolites in Two Rounds of Adventitious Root Formation in Softwood Cuttings of *Camellia sinensis* (L.). Agronomy.

[43] Chang E., Guo W., Dong Y., Jia Z.R., Zhao X.L., Jiang Z.P., Zhang L., Zhang J., Liu J.F. (2023). Metabolic profiling reveals key metabolites regulating adventitious root formation in ancient *Platycladus orientalis* cuttings. Front. Plant Sci..

[44] Petricka J.J., Winter C.M., Benfey P.N. (2012). Control of *Arabidopsis* Root Development. Annu. Rev. Plant Biol..

[45] Tong S., Kjaer J.E., Ogorek L.L.P., Pellegrini E., Song Z.W., Pedersen O., Herzog M. (2023). Responses of key root traits in the genus *Oryza* to soil flooding mimicked by stagnant, deoxygenated nutrient solution. J. Exp. Bot..

[46] Liu S.A., Sun Z.M., Xu M. (2018). Identification and characterization of long non-coding RNAs involved in the formation and development of poplar adventitious roots. Ind. Crops Prod..

[47] Wuyun T.N., Wang L., Liu H.M., Wang X.W., Zhang L.S., Bennetzen J.L., Li T.Z., Yang L.R., Liu P.F., Du L.Y. (2018). The Hardy Rubber Tree Genome Provides Insights into the Evolution of Polyisoprene Biosynthesis. Mol. Plant.

[48] Du Q.X., Wu Z.X., Liu P.F., Qing J., He F., Du L.Y., Sun Z.Q., Zhu L.L., Zheng H.C., Sun Z.Y. (2023). The chromosome-level genome of *Eucommia ulmoides* provides insights into sex differentiation and a-linolenic acid biosynthesis. Front. Plant Sci..

[49] Legué V., Rigal A., Bhalerao R.P. (2014). Adventitious root formation in tree species: Involvement of transcription factors. Physiol. Plant..

[50] Díaz-Sala C. (2014). Direct reprogramming of adult somatic cells toward adventitious root formation in forest tree species: The effect of the juvenile-adult transition. Front. Plant Sci..

[51] Vielba J.M., Vidal N., José M.C.S., Rico S., Sánchez C. (2020). Recent Advances in Adventitious Root Formation in Chestnut. Plants.

[52] Ahkami A.H., Lischewski S., Haensch K.T., Porfirova S., Hofmann J., Rolletschek H., Melzer M., Franken P., Hause B., Druege U. (2009). Molecular physiology of adventitious root formation in *Petunia hybrida* cuttings: Involvement of wound response and primary metabolism. New Phytol..

[53] Ahkami A., Scholz U., Steuernagel B., Strickert M., Haensch K.T., Druege U., Reinhardt D., Nouri E., von Wirén N., Franken P. (2014). Comprehensive Transcriptome Analysis Unravels the Existence of Crucial Genes Regulating Primary Metabolism during Adventitious Root Formation in *Petunia hybrida*. PLoS ONE.

[54] Rasmussen A., Hosseini S.A., Hajirezaei M.R., Druege U., Geelen D. (2015). Adventitious rooting declines with the vegetative to reproductive switch and involves a changed auxin homeostasis. J. Exp. Bot..

[55] Ruan J.J., Zhou Y.X., Zhou M.L., Yan J., Khurshid M., Weng W.F., Cheng J.P., Zhang K.X. (2019). Jasmonic Acid Signaling Pathway in Plants. Int. J. Mol. Sci..

[56] Ghorbel M., Brini F., Sharma A., Landi M. (2021). Role of jasmonic acid in plants: The molecular point of view. Plant Cell Rep..

[57] Ali M.S., Baek K.H. (2020). Jasmonic Acid Signaling Pathway in Response to Abiotic Stresses in Plants. Int. J. Mol. Sci..

[58] Waadt R., Seller C.A., Hsu P.K., Takahashi Y., Munemasa S., Schroeder J. (2022). Plant hormone regulation of abiotic stress responses. Nat. Rev. Mol. Cell Biol..

[59] Liu H., Timko M.P. (2021). Jasmonic Acid Signaling and Molecular Crosstalk with Other Phytohormones. Int. J. Mol. Sci..

[60] Sendon P.M., Seo H.S., Song J.T. (2011). Salicylic Acid Signaling: Biosynthesis, Metabolism, and Crosstalk with Jasmonic Acid. J. Korean Soc. Appl. Biol. Chem..

[61] Liu L.J., Sonbol F.M., Huot B., Gu Y.N., Withers J., Mwimba M., Yao J., He S.Y., Dong X.N. (2016). Salicylic acid receptors activate jasmonic acid signalling through a non-canonical pathway to promote effector-triggered immunity. Nat. Commun..

[62] Jang G., Yoon Y., Choi Y.D. (2020). Crosstalk with Jasmonic Acid Integrates Multiple Responses in Plant Development. Int. J. Mol. Sci..

[63] Lakehal A., Chaabouni S., Cavel E., Le Hir R., Ranjan A., Raneshan Z., Novák O., Pacurar D.I., Perrone I., Jobert F. (2019). A Molecular Framework for the Control of Adventitious Rooting by TIR1/AFB2-Aux/IAA-Dependent Auxin Signaling in *Arabidopsis*. Mol. Plant.

[64] Liu R., Wen S.S., Sun T.T., Wang R., Zuo W.T., Yang T., Wang C., Hu J.J., Lu M.Z., Wang L.Q. (2022). *PagWOX11/12a* positively regulates the *PagSAUR36* gene that enhances adventitious root development in poplar. J. Exp. Bot..

[65] Wang R.R., Bai T.H., Gao H.Y., Cui Y.J., Zhou R.L., Wang Z.Y., Song S.W., Jiao J., Wang M.M., Wan R. (2023). Genome-wide identification of *LBD* transcription factors in apple and the function of *MdLBD16a* in adventitious rooting and callus development. Sci. Hortic..

[66] Okushima Y., Fukaki H., Onoda M., Theologis A., Tasaka M. (2007). ARF7 and ARF19 Regulate Lateral Root Formation via Direct Activation of *LBD*/*ASL* Genes in *Arabidopsis*. Plant Cell.

[67] Agrawal R., Singh A., Giri J., Magyar Z., Thakur J.K. (2023). MEDIATOR SUBUNIT17 is required for transcriptional optimization of root system architecture in *Arabidopsis*. Plant Physiol..

[68] Feng K., Hou X.L., Xing G.M., Liu J.X., Duan A.Q., Xu Z.S., Li M.Y., Zhuang J., Xiong A.S. (2020). Advances in *AP2/ERF* super-family transcription factors in plant. Crit. Rev. Biotechnol..

[69] Neogy A., Garg T., Kumar A., Dwivedi A.K., Singh H., Singh U., Singh Z., Prasad K., Jain M., Yadav S.R. (2019). Genome-Wide Transcript Profiling Reveals an Auxin-Responsive Transcription Factor, *OsAP2/ERF-40*, Promoting Rice Adventitious Root Development. Plant Cell Physiol..

[70] Zhu H.C., Li C., Gao C.X. (2020). Applications of CRISPR-Cas in agriculture and plant biotechnology. Nat. Rev. Mol. Cell Bio..

[71] Wang P., Ma L.L., Li Y., Wang S.A., Li L.F., Yang R.T., Ma Y.Z., Wang Q. (2016). Transcriptome profiling of indole-3-butyric acid-induced adventitious root formation in softwood cuttings of the Catalpa bungei variety ‘YU-1’ at different developmental stages. Genes Genom..

[72] Munir M.Z., Din S.U., Imran M., Zhang Z.J., Pervaiz T., Han C., Nisa Z.U., Bakhsh A., Muneer M.A., Sun Y.H. (2021). Transcriptomic and Anatomic Profiling Reveal Etiolation Promotes Adventitious Rooting by Exogenous Application of 1-Naphthalene Acetic Acid in *Robinia pseudoacacia* L.. Forests.

[73] Mobin M., Wu C.H., Tewari R.K., Paek K.Y. (2015). Studies on the glyphosate-induced amino acid starvation and addition of precursors on caffeic acid accumulation and profiles in adventitious roots of *Echinacea purpurea* (L.) Moench. Plant Cell Tiss. Org..

[74] Koramutla M.K., Tuan P.A., Ayele B.T. (2022). Salicylic Acid Enhances Adventitious Root and Aerenchyma Formation in Wheat under Waterlogged Conditions. Int. J. Mol. Sci..

[75] Cheniany M., Ganjeali A. (2016). Developmental role of phenylalanine-ammonia-lyase (*pal*) and cinnamate 4-hydroxylase (*c4h*) genes during adventitious rooting of juglans regia l. microshoots. Acta Biol. Hung..

[76] Tian Y.Y., Bai S.L.G., Dang Z.H., Hao J.F., Zhang J., Hasi A. (2019). Genome-wide identification and characterization of long non-coding RNAs involved in fruit ripening and the climacteric in *Cucumis melo*. BMC Plant Biol..

[77] Engreitz J.M., Haines J.E., Perez E.M., Munson G., Chen J., Kane M., McDonel P.E., Guttman M., Lander E.S. (2016). Local regulation of gene expression by lncRNA promoters, transcription and splicing. Nature.

[78] Long Y.C., Wang X.Y., Youmans D.T., Cech T.R. (2017). How do lncRNAs regulate transcription?. Sci. Adv..

[79] Patturaj M., Munusamy A., Kannan N., Ramasamy Y. (2022). Biologia Futura: Progress and future perspectives of long non-coding RNAs in forest trees. Biol. Futur..

[80] Chekanova J.A. (2015). Long non-coding RNAs and their functions in plants. Curr. Opin. Plant Biol..

[81] Hou J.N., Lu D.D., Mason A.S., Li B.Q., Xiao M.L., An S.F., Fu D.H. (2019). Non-coding RNAs and transposable elements in plant genomes: Emergence, regulatory mechanisms and roles in plant development and stress responses. Planta.

[82] Ran N., Liu S., Qi H.R., Wang J.L., Shen T.F., Xu W.L., Xu M. (2023). Long Non-Coding RNA lncWOX11a Suppresses Adventitious Root Formation of *Poplar* by Regulating the Expression of *PeWOX11a*. Int. J. Mol. Sci..

[83] Qi H.R., Wu L., Shen T.F., Liu S., Cai H., Ran N., Wang J.L., Xu M. (2023). Overexpression of the long non-coding RNA lncWOX5 negatively regulates the development of adventitious roots in *Populus*. Ind. Crops Prod..

[84] Um Y., Lee Y., Kim S.C., Jeong Y.J., Kim G.S., Choi D.W., Cha S.W., Kim O.T. (2017). Expression analysis of ginsenoside biosynthesis-related genes in methyl jasmonate-treated adventitious roots of *Panax ginseng* via DNA microarray analysis. Hortic. Environ. Biotechnol..

[85] Zhao X.H., Zhao Y.J., Gou M.Y., Liu C.J. (2023). Tissue-preferential recruitment of electron transfer chains for cytochrome P450-catalyzed phenolic biosynthesis. Sci. Adv..

[86] Hansen C.C., Nelson D.R., Moller B.L., Werck-Reichhart D. (2021). Plant cytochrome P450 plasticity and evolution. Mol. Plant.

[87] Gou M.Y., Ran X.Z., Martin D.W., Liu C.J. (2018). The scaffold proteins of lignin biosynthetic cytochrome P450 enzymes. Nat. Plants.

[88] Alber A.V., Renault H., Basilio-Lopes A., Bassard J.E., Liu Z., Ullmann P., Lesot A., Bihel F., Schmitt M., Werck-Reichhart D. (2019). Evolution of coumaroyl conjugate 3-hydroxylases in land plants: Lignin biosynthesis and defense. Plant J..

[89] Guttikonda S.K., Trupti J., Bisht N.C., Chen H., An Y.Q.C., Pandey S., Xu D., Yu O. (2010). Whole genome co-expression analysis of soybean cytochrome P450 genes identifies nodulation-specific P450 monooxygenases. BMC Plant Biol..

[90] Babu P.R., Rao K.V., Reddy V.D. (2013). Structural organization and classification of cytochrome P450 genes in flax (*Linum usitatissimum* L.). Gene.

[91] Lin C., Ogorek L.L.P., Liu D., Pedersen O., Sauter M. (2023). A quantitative trait locus conferring flood tolerance to deepwater rice regulates the formation of two distinct types of aquatic adventitious roots. New Phytol..

[92] Da Costa C.T., de Almeida M.R., Ruedell C.M., Schwambach J., Maraschin F.S., Fett-Neto A.G. (2013). When stress and development go hand in hand: Main hormonal controls of adventitious rooting in cuttings. Front. Plant Sci..

[93] Kim D., Paggi J.M., Park C., Bennett C., Salzberg S.L. (2019). Graph-based genome alignment and genotyping with HISAT2 and HISAT-genotype. Nat. Biotechnol..

[94] Pertea M., Pertea G.M., Antonescu C.M., Chang T.C., Mendell J.T., Salzberg S.L. (2015). StringTie enables improved reconstruction of a transcriptome from RNA-seq reads. Nat. Biotechnol..

[95] Love M.I., Huber W., Anders S. (2014). Moderated estimation of fold change and dispersion for RNA-seq data with DESeq2. Genome Biol..

[96] Chen C., Wu Y., Li J., Wang X., Zeng Z., Xu J., Liu Y., Feng J., Chen H., He Y. (2023). TBtools-II: A “One for All, All for One” Bioinformatics Platform for Biological Big-data Mining. Mol. Plant.

[97] Yu G.C., Wang L.G., Han Y.Y., He Q.Y. (2012). clusterProfiler: An R Package for Comparing Biological Themes Among Gene Clusters. Omics.

[98] Chen T., Liu Y.X., Huang L. (2022). ImageGP: An easy-to-use data visualization web server for scientific researchers. iMeta.

[99] Lu S.N., Wang J.Y., Chitsaz F., Derbyshire M.K., Geer R.C., Gonzales N.R., Gwadz M., Hurwitz D.I., Marchler G.H., Song J.S. (2020). CDD/SPARCLE: The conserved domain database in 2020. Nucleic Acids Res..

[100] Shannon P., Markiel A., Ozier O., Baliga N.S., Wang J.T., Ramage D., Amin N., Schwikowski B., Ideker T. (2003). Cytoscape: A Software Environment for Integrated Models of Biomolecular Interaction Networks. Genome Res..

[101] Emms D.M., Kelly S. (2019). OrthoFinder: Phylogenetic orthology inference for comparative genomics. Genome Biol..

