# Supplementary material for: Integrated Transcriptomics and Metabolomics Analysis Promotes the Understanding of Adventitious Root Formation in Eucommia ulmoides Oliver
Source: Plants (Basel). 2024 Jan 3;13(1):136. doi: 10.3390/plants13010136 (PMC10780705; doi:10.3390/plants13010136)
Supplement: Supplementary file 1 [file plants-13-00136-s001.zip › plants-2792696-supplementary.pdf]

Table S1. Summary of strand-specific RNA sequencing data

Sample	ReadSum	BaseSum	Q20 (%)	Q30 (%)	GC content (%)
H6S1_1	54,894,571	16,414,120,460	98.53	95.75	45.72
H6S1_2	53,964,718	16,011,007,144	98.13	94.47	45.21
H6S2_1	55,544,404	16,556,997,312	98.56	95.88	46.94
H6S2_2	52,580,184	15,671,930,776	97.97	94.09	49.16
H6S3_1	62,026,829	18,526,292,102	97.98	94.23	46.31
H6S3_2	53,966,808	16,057,917,090	98.19	94.7	47.09
H8S1_1	54,715,454	16,318,408,714	98.25	95.03	43.84
H8S1_2	62,438,833	18,647,704,660	98.06	94.23	44.2
H8S2_1	61,910,827	18,519,149,868	98.03	94.17	43.12
H8S2_2	63,919,993	19,102,954,842	98.06	94.24	43.31
H8S3_1	56,674,564	16,893,155,158	98.28	95.13	44.23
H8S3_2	56,602,301	16,876,900,682	97.88	94.2	45.17

Table S2. *E. ulmoides* hub genes in blue module and turquoise module and *A. thaliana* orthologous genes

Modules	***E. ulmoides*** **hub genes**	***A. thaliana*** **orthologous genes**
blue	*EU0125382*	*AT1G19840*, *AT1G75590*, *AT5G10990*
blue	*EU0121826*	*AT1G02330*, *AT5G28470*
turquoise	*EU0114710*	*ATCG00860*, *ATCG01280*
turquoise	*EU0128499*	*AT1G50680*, *AT1G51120*
turquoise	*EU0104849*	*AT1G35880*, *AT3G46740*
blue	*EU0109380*	*AT5G48380*
blue	*EU0100319*	*AT1G45474*
blue	*EU0117006*	*AT1G71340*
blue	*EU0109832*	*AT2G26210*
blue	*EU0106969*	*AT3G27960*
turquoise	*EU0107185*	*AT1G13680*
turquoise	*EU0125430*	*AT1G49320*
turquoise	*EU0112695*	*AT2G26690*
turquoise	*EU0127605*	*AT3G02510*
turquoise	*EU0124232*	*AT5G06080*
blue	*EU0100375*	none
blue	*EU0103658*	none
blue	*EU0105208*	none
blue	*EU0113347*	none
blue	*EU0120751*	none
blue	*EU0105844*	none
blue	*EU0109382*	none
blue	*EU0109584*	none
blue	*EU0109541*	none
blue	*EU0111608*	none
blue	*EU0116878*	none
blue	*EU0120981*	none
blue	*EU0129154*	none
turquoise	*EU0116224*	none
turquoise	*EU0113433*	none
turquoise	*EU0127354*	none
turquoise	*EU0117900*	none
turquoise	*EU0116412*	none
turquoise	*EU0110081*	none
turquoise	*EU0119332*	none
turquoise	*EU0113897*	none
turquoise	*EU0100696*	none
turquoise	*EU0102912*	none
turquoise	*EU0117354*	none
turquoise	*EU0131180*	none

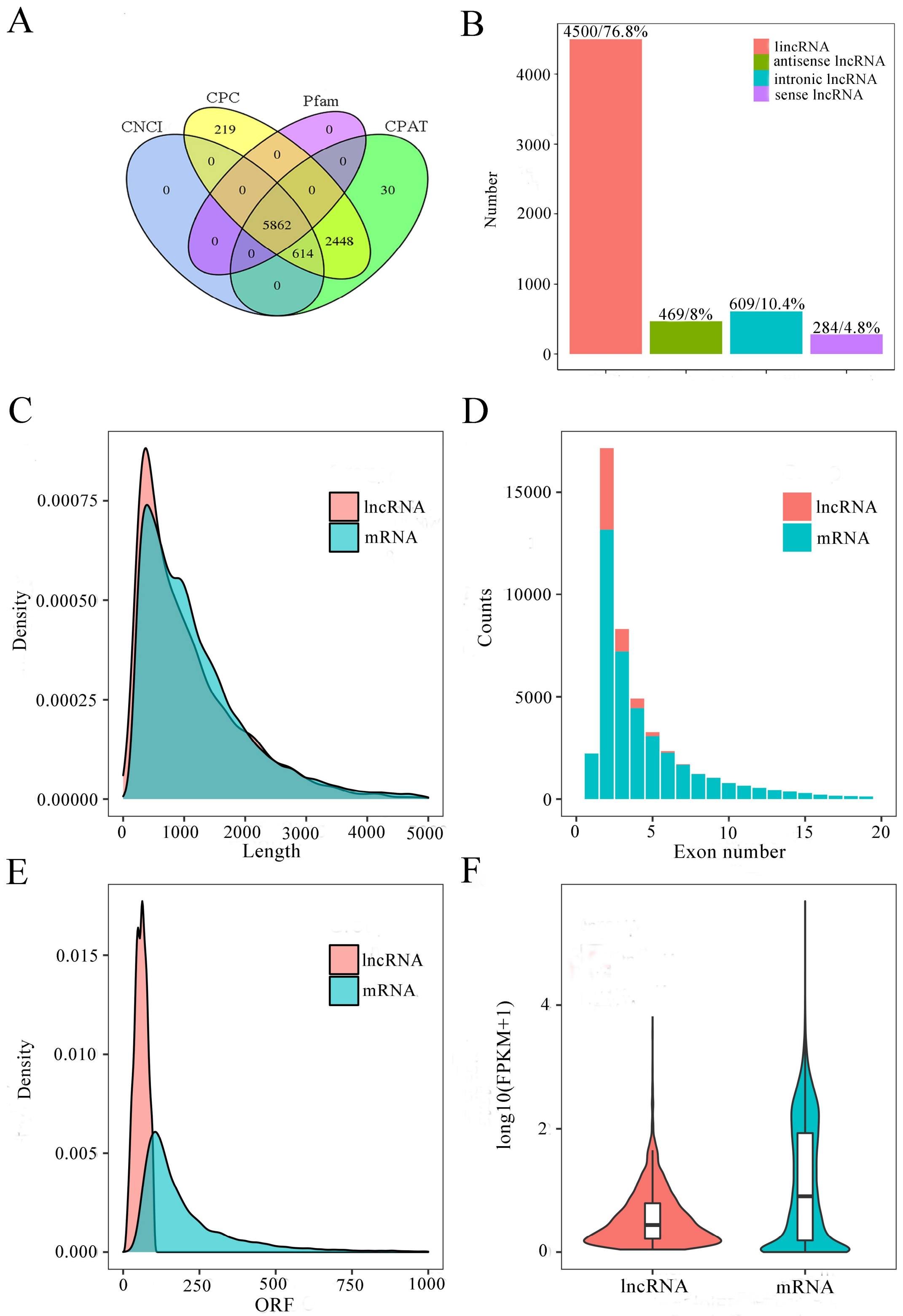

Figure. S1 The basic characteristics of lncRNA and mRNA during *E. ulmoides* AR development. (A) Screening results by CNCI, CPC, Pfam and CPAT. (B) Classification of different types of lncRNAs. (C) Distribution of transcript length. (D) Distribution of the number of exons. (E) Distribution of ORFs. (F) Comparison of expression levels.

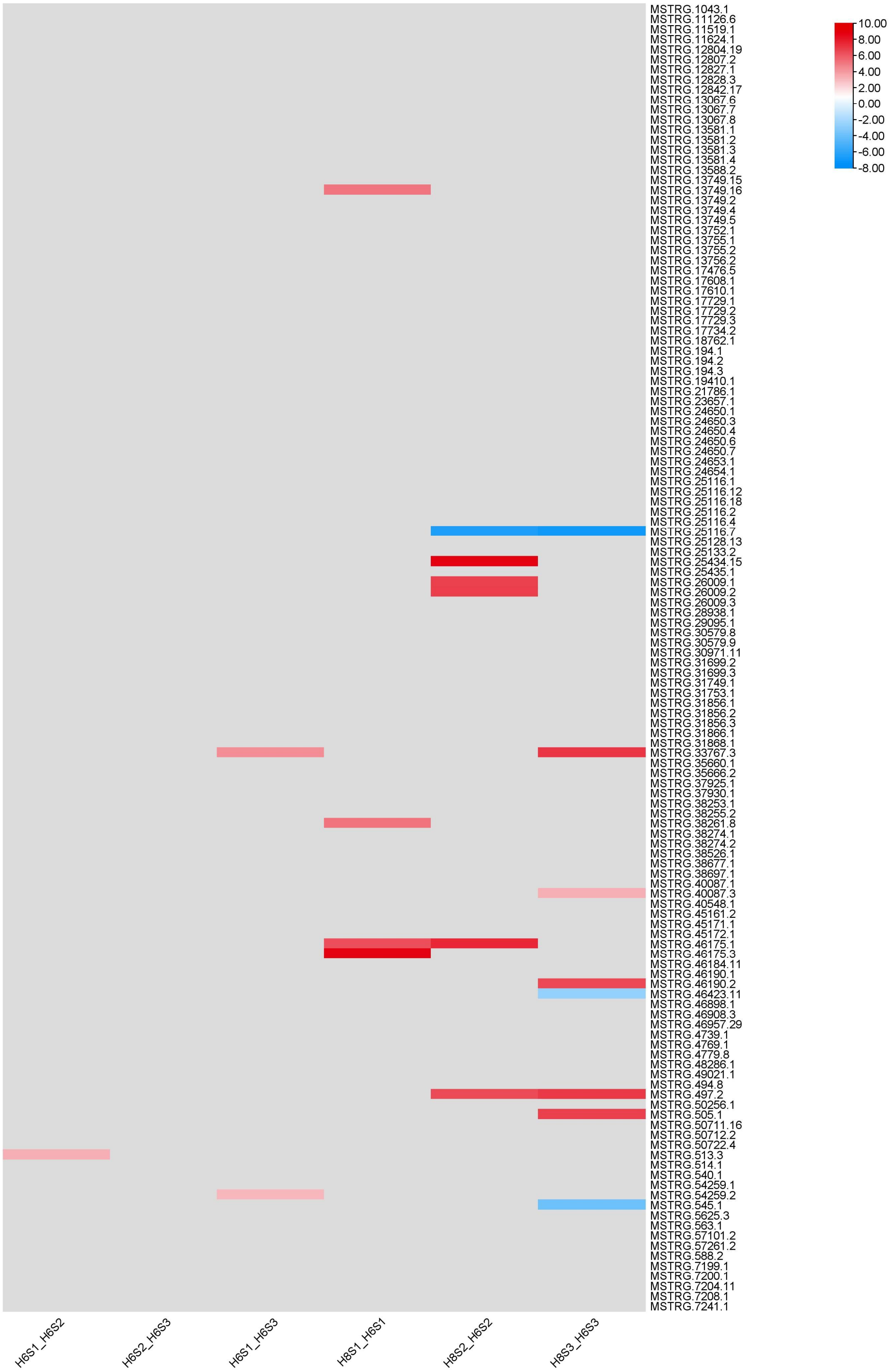

Figure. S2 The differential expression of 130 *cis*-acting lncRNAs during cutting rooting in favorable growth environment. The red boxes, blue boxes and grey boxes represent significant up-regulation, significant down-regulation and no significant change, respectively. The color depth of red boxes and blue boxes represents the size of $\log_2$ fold change.
